# Non-invasively accuracy enhanced blood glucose sensor using shallow dense neural networks with NIR monitoring and medical features

**DOI:** 10.1038/s41598-022-05570-8

**Published:** 2022-02-02

**Authors:** Chavis Srichan, Wachirun Srichan, Pobporn Danvirutai, Chanachai Ritsongmuang, Amod Sharma, Sirirat Anutrakulchai

**Affiliations:** 1 Faculty of Engineering, Khon Kaen University, Khon Kaen, Thailand; 2 Thakhantho Hospital, Kalasin, Thailand; 3 T. Robotics, Co. Ltd., Bangkok, Thailand; 4 Bureau of Academic Services, Khon Kaen University, Khon Kaen, Thailand; 5 Chronic Kidney Disease Prevention in the Northeast of Thailand (CKDNET), Khon Kaen University, Khon Kaen, Thailand; 6 Faculty of Medicine, Khon Kaen University, Khon Kaen, Thailand

**Keywords:** Biomedical engineering, Engineering

## Abstract

Non-invasive and accurate method for continuous blood glucose monitoring, the self-testing of blood glucose is in quest for better diagnosis, control and the management of diabetes mellitus (DM). Therefore, this study reports a multiple photonic band near-infrared (mbNIR) sensor augmented with personalized medical features (PMF) in Shallow Dense Neural Networks (SDNN) for the precise, inexpensive and pain free blood glucose determination. Datasets collected from 401 blood samples were randomized and trained with ten-fold validation. Additionally, a cohort of 234 individuals not included in the model training set were investigated to evaluate the performance of the model. The model achieved the accuracy of 97.8% along with 96.0% precision, 94.8% sensitivity and 98.7% specificity for DM classification based on a diagnosis threshold of 126 mg/dL for diabetes in fasting blood glucose. For non-invasive real-time blood glucose monitoring, the model exhibited ± 15% error with 95% confidence interval and the detection limit of 60–400 mg/dL, as validated with the standard hexokinase enzymatic method for glucose estimation. In conclusion, this proposed mbNIR based SDNN model with PMF is highly accurate and computationally cheaper compared to similar previous works using complex neural network. Some groups proposed using complicated mixed types of sensors to improve noninvasive glucose prediction accuracy; however, the accuracy gain over the complexity and costs of the systems harvested is still in questioned (Geng et al. in Sci Rep 7:12650, 2017). None of previous works report on accuracy enhancement of NIR/NN using PMF. Therefore, the proposed SDNN over PMF/mbNIR is an extremely promising candidate for the non-invasive real-time blood glucose monitoring with less complexity and pain-free.

## Introduction

Diabetes Mellitus (DM) is deteriorating overall health situation throughout the globe. Conditions associated with the disease particularly cardiovascular, neurological and nephrological complications has exerted immense burden in terms of cost and the quality of life in patients^1^. It has been reported that individuals with diabetes have double risk of all-cause mortality^2^. Therefore, an early prognosis and control of DM is inevitable to prevent severity and disease progression. A standard guideline for the diagnosis of DM is a fasting blood sugar level of 126 mg/dL (7 mmol/L) or higher on two separate tests^3^ and a continuous blood glucose monitoring is vital for the adjustment of lifestyle and diabetes management. Currently, dextrostrix is popular for blood glucose measurement in DM patients; however, the method has some disadvantages such as invasive, non real-time and expensive.

**Figure 1:**
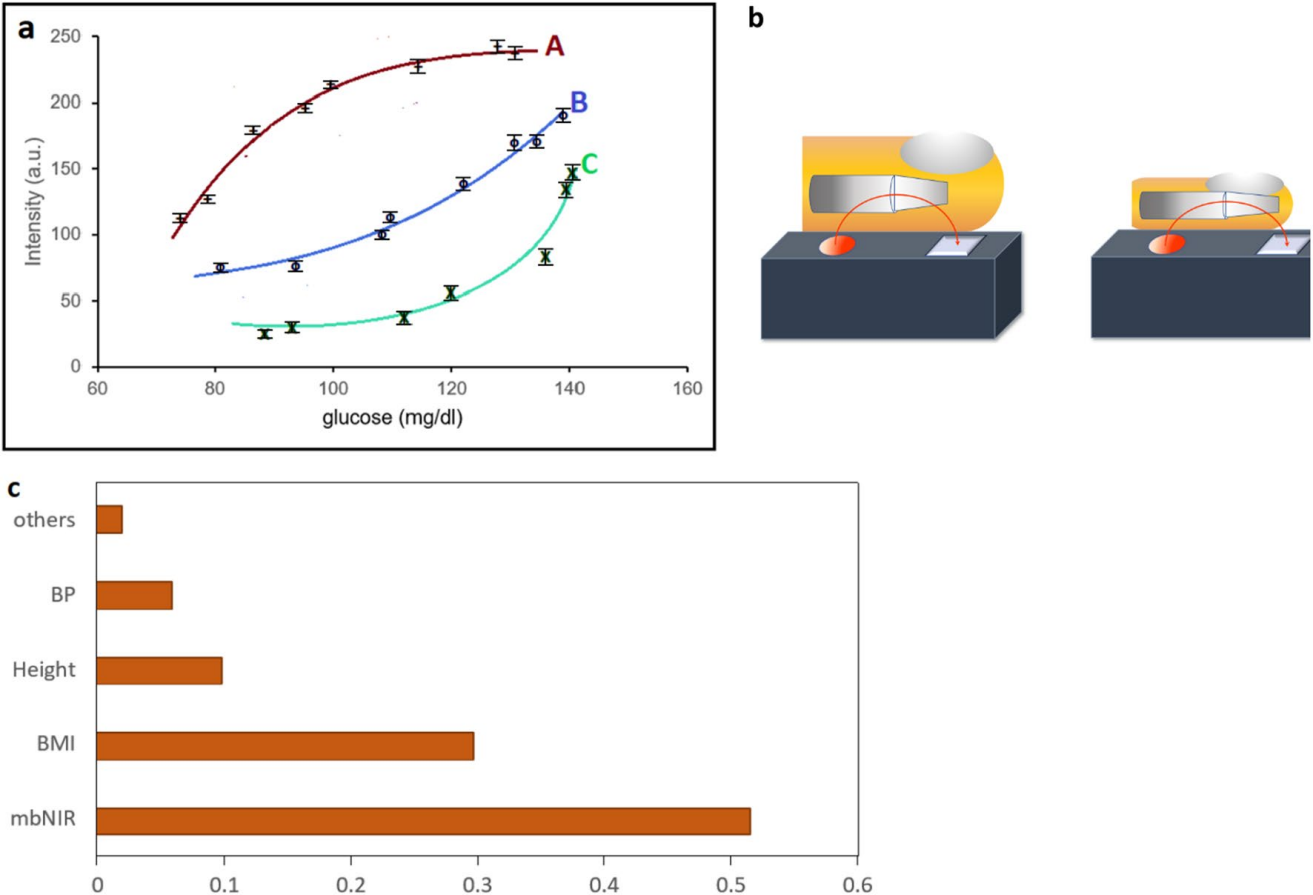
(**a**) A graph showing NIR readout and blood glucose of two subjects with different PMF. For persons A, B and C we obtained different glucose curves without PMF information. Therefore, we imposed PMF into SDNN to predict non-invasive blood glucose with significantly improvement in accuracy. (**b**) Illustrate different bones and skin thickness which contribute the difference in NIR vs glucose curves (**c**) feature importance of each parameter.

**Figure 2:**
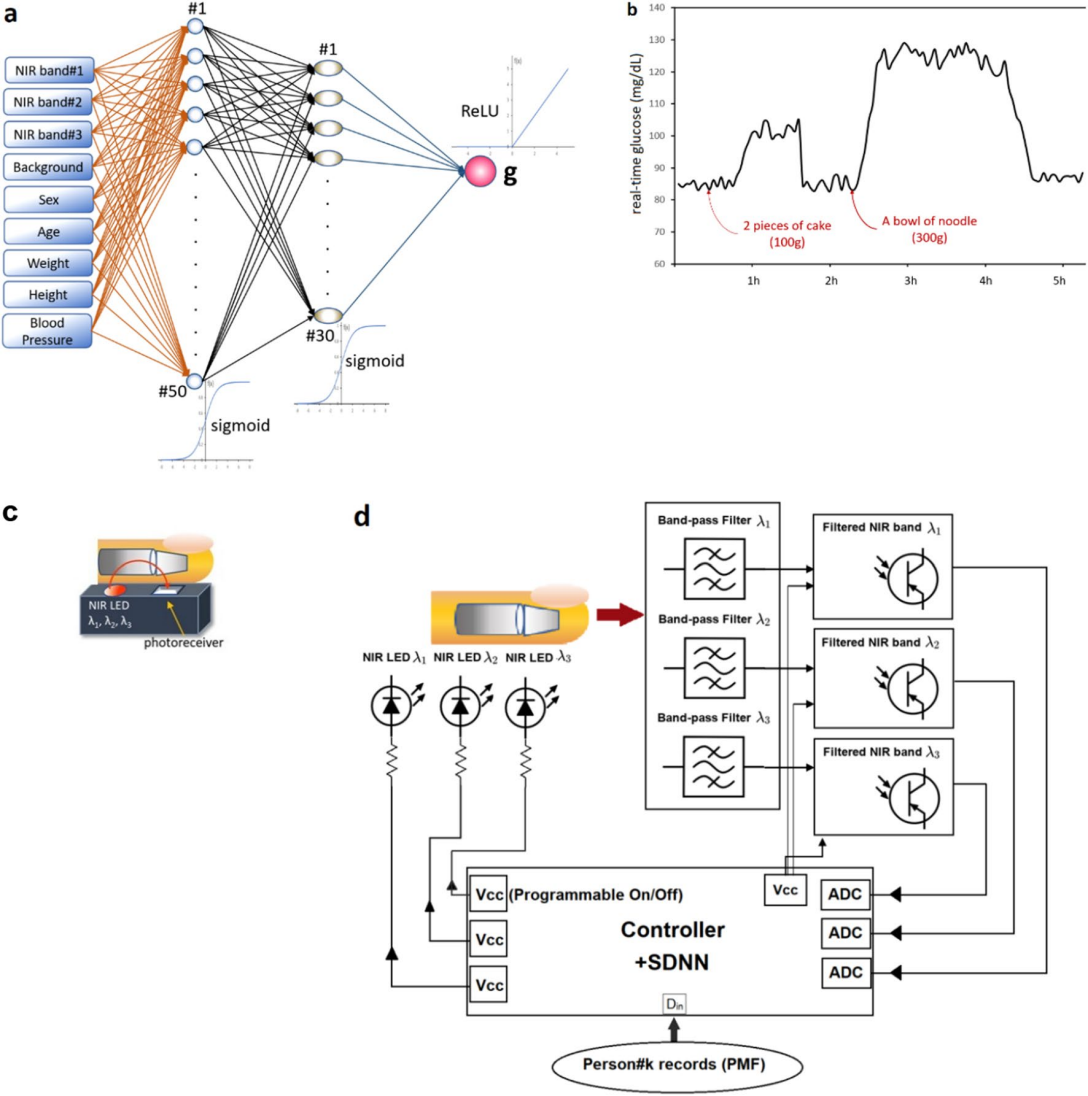
Our real-time glucose monitoring (**a**) the overall framework (**b**) experiment on real usage in a person (low-pass filtered) Shallow Dense Neural Networks using mbNIR and PMF as input data for DM classification and Real-time Blood Glucose Monitoring (**a**) mbNIR non-invasive measurement, (**b**) glucose absorbance spectrum (**c**) brief alignment between finger and sensor and (**d**) Schematic of the mbNIR/PMF + SDNN illustrating that light scattered from finger was filtered before entering readout channel of the Controller, for band#1 (800–900 nm) centered at 850 nm, band#2 (900–1000 nm) centered at 950 nm, and band#3 (1100–1200 nm) centered at 1150 nm respectively.

Recently, there are Medtronic/MiniMed, Dexcom, and Abbott which are continuous blood glucose monitoring systems which measure blood glucose fluctuation in interstitial fluid. All of these methods are still invasive and suffer from short-term noise^4^. The noise occurred in the system will cause the drop in accuracy significantly and the system need daily calibration due to its error fluctuations and these were invasive. In this work, we proposed an SDNN model over mbNIR and PMF for real-time blood glucose monitoring non-invasively. Our work could predict the relationship between blood glucose concentration using mbNIR and PMF which is shown in Figs. 1 and 2.

With the advancement of artificial intelligence, or in general Artificial Neural Networks (ANNs). ANNs is a type of supervised machine learning used to model a system where an explicit equation cannot be formed to precisely calculate the output. ANNs are composed of multiple-layer perceptrons whose weights of summation were trained to minimize prediction error of a model. Hybridizing ANNs into biological and physical features could enhance glucose sensor accuracy. ANN is used to construct function of multiple variables where the multiple inputs and the output can be adjust to maximize accuracy by training the weights in the hidden layer nodes. The model comprises of sequential perceptron nodes connected between layers by adjustable weights. All Convolutional Neural Networks (CNNs), Deep Neural Networks (DNNs) and SDNNs are subset of ANNs. CNNs and DNNs employ a large number of hidden layers which multiply the complexity of computation. SDNN uses a few hidden layers with increased numbers of nodes in each hidden layers (dense); the total number of perceptrons and the depth is significantly less than those of CNNs and DNNs. This results in less computational complexity in SDNNs.

Non-invasive approaches are being developed as an ideal candidate for non-invasive, low-cost and the frequent testing of glucose. One of non-invasive techniques employed for glucose monitoring is absorbance spectroscopy, which detects glucose by its optical signature. Published literatures suggest that there has been some attempt to construct glucose sensor by introducing near-infrared (NIR) glucose sensing but these used only single or multiple-band NIR (mbNIR) to train Neural Networks (NN), and thereby generated insufficient accuracy^5^. Carno-Garcia et al. (2021) reported using combination of NIR and 37–39 GHz frequency to excite and measure response for glucose determination together with ANN; however, the experiment was done in aquas solution, in vitro^6^.

Apart from using NIR, other approaches, e.g. ring-resonator was proposed as a non-invasive glucose sensing^7,8^. Bent et al. used smartwatches with food consumption records to track glucose^9^. Several methods were proposed for non-invasive glucose sensing; however, none of them could achieve both high accuracy and low cost at the same time. All of methods mentioned above still suffers from the interference problem due to personal characteristics of bones, tissues, fat, humidity^10^. The main concern in previously reported models were the lack of personalized medical features (PMF) for the training of networks. It is to highlight that an interference due to bones, tissue thickness, fat and other unknown factors causes low accuracy because of interference in NIR response as shown in Fig. 1b, and for that reason, an inclusion of PMF could be advantageous to overcome individual discrepancies^11,12^.

Beer-Lambert law relates light attenuation and the properties of medium. In theory, the concentration of absorbing species is proportional to the absorbance in the reactive wavelength^13,14^. However, different personalized characteristics of tissues, fat, bones, and thickness. affect the intensities of multiple-band NIR scattering. In Beer-Lambert theory, the transmittance (*T*) was expressed by^15^

1 $$\exp \left( {\mathop \sum \limits_{i = 1}^{n} \sigma_{i} \mathop \smallint \limits^{{l_{i} }} n_{i} \left( x \right)dx} \right) = \exp \left( { - \sum \tau_{i} } \right),$$

where $\sigma_{i}$ is the attenuation cross section, $n_{i}$ is the density of attenuating species or concentration,

$\tau_{i}$ is the absorbance for species *i* and *l*_*i*_ is the path length of beam light for species *i*. When blood glucose concentration is high, the law would break down if the material is highly scattering. Assume uniform *n*_*i*_* over* spatial domain,

2 $$T = {\text{exp}}\left( { - \mathop \sum \limits_{i = 1}^{n} \sigma_{i} n_{i} l_{i} } \right).$$

For each person *k*,

3 $$T^{k} = {\text{exp}}\left( { - \mathop \sum \limits_{i = 1}^{n} \sigma_{i} n_{i}^{k} l_{i}^{k} } \right).$$

For many species such as blood tissue, bones, water, fat, it would be impossible to fit all the parameters. Therefore, we suppose that *σ*_*g*_ and *n*_*g*_ represent attenuation cross section and density or concentration of blood glucose for person *k:*

4 $$n_{g} = \left[ {\ln \left( {T^{k} } \right) - \mathop \sum \limits_{i} \sigma_{i} n_{i}^{k} l_{i}^{k} } \right]/\left( {\sigma_{g} l_{g}^{k} } \right),$$

5 $$n_{g}^{k} = \ln \left( {T^{k} } \right)/\sigma_{g} l_{g}^{k} - \mathop \sum \limits_{i} (\sigma_{i} n_{i}^{k} l_{i}^{k} )/\left( {\sigma_{g} l_{g}^{k} } \right).$$

Define relative attenuation cross section and relative light beam path of species i to glucose respectively

6 $$\sigma_{ig} = \sigma_{i} /\sigma_{g} ,$$

7 $$l_{ig}^{k} = l_{i}^{k} /l_{g}^{k}$$

where the subscript *g* denote glucose. The glucose concentration of the person *k* were then expressed by

8 $$n_{g}^{k} = \frac{{\ln \left( {T^{k} } \right)}}{{\sigma_{g} l_{g}^{k} }} - \mathop \sum \limits_{i} \sigma_{ig} l_{ig}^{k} n_{i}^{k} .$$

In order to determine $n_{g}^{k}$ or glucose concentration of person *k*, one has many unknown parameters {$\sigma_{ig} , l_{ig}^{k} , n_{i}^{k} , l_{g}^{k}$} let $l_{ig}^{k} n_{i}^{k} = N_{i}^{k}$,

9 $$n_{g}^{k} = {\text{ln}}\left( {T^{k} } \right)/\sigma_{g} l_{g}^{k} - \sigma_{ig} N_{i}^{k} .$$

Assume that $N_{i}^{k}$ is a function of PMF(gender, weight, BMI, BP, age, …), i.e.,

10 $$N_{i}^{k} = \left( {f_{1}^{k} ,f_{2}^{k} , f_{3}^{k} , \ldots ,f_{p}^{k} } \right) = F^{k} ,$$

The main purpose was to illustrate the infeasibility of using analytical prediction due to unpredictable scattering and absorption of the blood glucose, since the absorption/scattering properties changed. Low-enough blood glucose could be absorbed in relation with the glucose concentration but for high concentration blood glucose. Explanations were added to the paragraph after Eq. 9 (as highlighted in green color). Another purpose is to introduce using shallow dense neural network to estimate the function between PMF and mbNIR and the expected glucose level.

Since $\sigma_{{{\text{HbO}}_{2} }}$ and $\sigma_{{{\text{HbR}}}}$ which are the absorption terms of the oxygenated hemoglobin (HbO_2_) and de-oxygenated hemoglobin (HbR) respectively in the wavelength that we use will be three orders of magnitude smaller than $\sigma_{{\text{g}}}$ according to Kong et al. (2013)^16^. Hence the total terms including HbO_2_ and HbR in this form $\sigma_{{i\left\{ {HbO2} \right\}}} l_{{i\left\{ {HbO2} \right\}}}^{k} n_{i}^{k}$ and $\sigma_{{i\left\{ {HbR} \right\}}} l_{{i\left\{ {HbR} \right\}}}^{k} n_{i}^{k}$ (Eq. 8) are negligible compared to the glucose species scattering/absorption at NIR wavelength^15^. Referring to Eq. (9), the term $\sigma_{ig} N_{i}^{k}$ is a function of PMF and *F*^*k*^ varies for three persons in the experiment as shown in Fig. 1a. All of these terms could not be estimated directly using regression analysis for each person simultaneously because of the scattering and absorption term will be different for each blood glucose level as well as other absorbing and scattering species in different person resulting in infeasibility of constructing the blood glucose model using Eq. (9). Therefore, it is possible to use artificial neural network instead in order to construct the non-invasive glucose prediction model using mbNIR and PMF over SDNN numerically for each person. The resulting performance as well as feature importance was shown in the Results section.

The relationship will be simple if the concentration of all species is low enough and all species are all absorbed. However, in many cases, for some time *t*, person *k* could develop high concentration of $n_{g}^{k}$ and results in changing sign of $\sigma_{g}$ and $\sigma_{ig}$ as the scattering dominates the process. Therefore, direct parameter fitting will be extremely complicated. In this case, we would use ANN to approximate $N_{l}^{k}$ of each person from the set $F^{k}$ and $T^{k}$ using datasets of $\left( {F^{k} , T^{k} } \right)$ of many persons. in order to predict *n*_*g*_ for a known person with (*F*, *T*).

In this work, feature F will be called PMF (Personalized Medical Feature). It will be augmented into mbNIR feature to improve accuracy of the shallow-dense neural network prediction for non-invasive glucose. There will be a turning point of the curve *T* vs *n*_*g*_ due to the scattering dominate at high *n*_*g*_ instead of absorption process which is shown in Fig. 1a.

This study proposed a new model of glucose monitoring using mbNIR and PMF for training the Shallow Dense Neural Networks (SDNN) possessing lower complexity compared to deep-structured networks^17^. Complex neural network such as Deep Neural Networks (DNNs) or Convolutional Neural Networks (CNNs) without PMF can increase an accuracy of a sensor model to a satisfied level^18^ but our more simple SDNN with PMF, the higher accuracy could be achieved. CNNs and DNNs without PMF were proven to have less accuracy and precision^18^. The design complexity was avoided in our current prototype. This is mainly due to the fact that DNNs and CNNs consume too much computational costs since the complexity of the model increase with the number of hidden layers or depth of the neural network^17^, which is not ultimate for small, embedded devices with the aim of low cost for people. Instead, the shallowness of NN layers using dense nodes inside could provide enhanced computational speed and improved accuracy without the requirement of deep layers. To our knowledge none of the absorbance-based glucose sensing model has used PMF along with SDNN for the improvement of accuracy and precision till date^18^.

## Results

### NIR non-invasive blood glucose prediction

Our initial experiment showed that two persons with different PMFs hold different curves between glucose and intensity as shown in Fig. 1a. An illustration of different personal characteristics was shown in Fig. 1b.

The feature importance could be found using the exclusion method. For each one of them, we exclude the feature that we would like to find the importance from the dataset and then compute the accuracy drop. The resulting feature importance could be concluded that combining personalized medical records involve feature importance of 0.48; while using mbNIR alone yield 0.52 importance. In Fig. 1c, the feature importance of PMF, i.e., BMI, height, weights, age and blood pressure contributed significantly (48%) compared to barely using mbNIR (52%). Therefore, imposing PMF into the SDNN model would significantly improve the accuracy of the non-invasive prediction.

### The SDNN model outline

Real-time non-invasive glucose monitoring: Fig. 2a illustrates the overall framework and Fig. 2b reports an experiment showing time series of non-invasive glucose values. It can be concluded that the predicted glucose levels are correlated with food consumption time. After the person taking 2 pieces of cake, glucose level increase by 20 and after taking a bowl of noodle, predicted glucose increased by 25–30 mg/dL. The decrement slope of glucose level could possibly be described by the functional response of insulin as a result of blood glucose increment (in non-diabetic human).

### Performance analysis of the model

**Figure 3:**
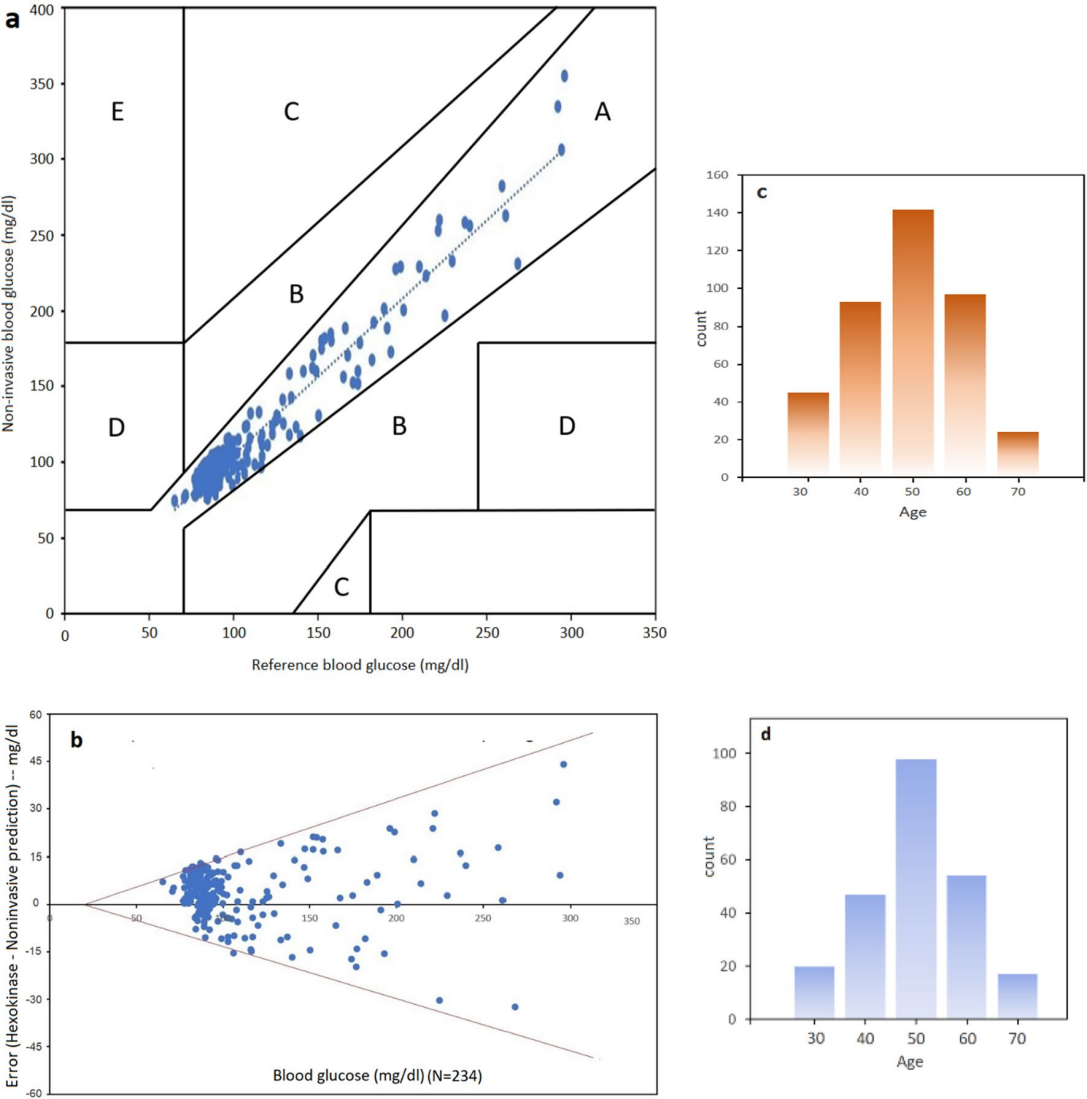
(**a**) Clark error grid plot illustrating all points are in region A (clinically accurate, +/− 20% error), (**b**) Bland–Altman (95% confidence plot). (**c**) Histogram illustrating age of training cohorts (N = 401) having median at 50 years. (**d**) Histogram of the testing group (N = 234).

The model was trained by previously collected 401 dataset including gender, age, weight, height and blood pressure as PMF. An accuracy of 98.5% along with 98.0% precision, 96.2% sensitivity and 99.3% specificity were achieved. Next, the model performance analysis performed in a cohort of 234 individuals not included in the training set, the testing prediction accuracy was 96.6% with 91.7% precision, 91.7% sensitivity and 97.8% specificity. These calculations were based on the fasting blood glucose level of 126 mg/dL, a diagnosis threshold for DM. The prediction of DM was done with a ten-fold cross validation of input data, when an error of ± 15% within 95% confidence level was recorded in the range of 60–400 mg/dL, as validated by standard Hexokinase method (Fig. 3a,b). There was merely 2–6% performance drop when the test set was compared with the training set.

**Figure 4:**
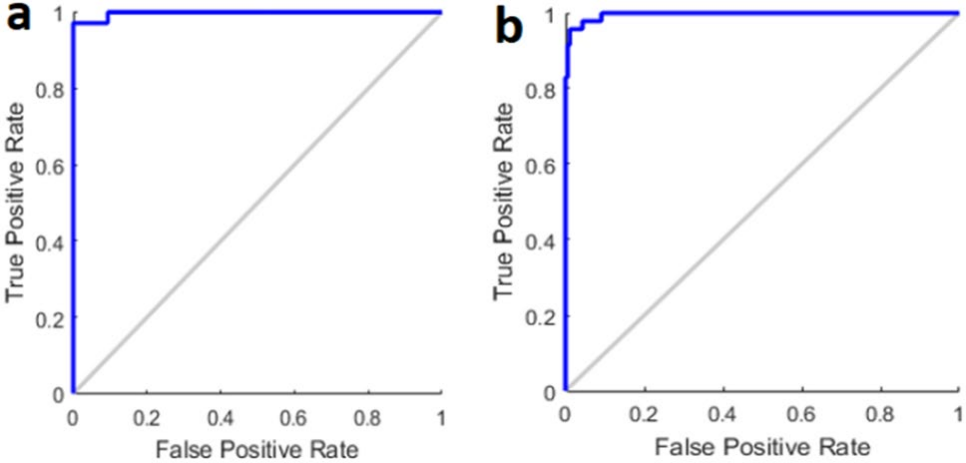
ROC plot in DM classification in (**a**) training data (**b**) test data.

For real-time blood glucose prediction, the data was plotted showing that at N = 234, samples fall in region “A” or clinically accurate according to Clarke Error Grid (CEG) analysis (Fig. 3a). In CEG, critical errors are marked by E and less critical errors are marked by D, C and B regions respectively. In addition, Bland–Altman plot was used as the statistical analysis of the experiments (Fig. 3b). Bland–Altman plot shows how the errors behave at 95% confidence level. The result was that mbNIR + PMF over SDNN yields error between +/− 15%. Bland–Altman is a generic and established statistical analysis for measuring the agreement between two methods. Usually, the first method is the gold standard reference, the second one is the method being proposed. Receiver Operating Characteristic Curve (ROC) for training and performance (test) set is shown in Fig. 4a,b. Moreover, the results of confusion matrices for DM prediction were shown in Table 1. Age distribution of cohorts in training and testing set were shown in Fig. 3c,d respectively.

**Table 1:** The results of confusion matrices for DM prediction (threshold at 126 mg/dl); (a) Training, N = 401 (b) test dataset, N = 234 (c) overall data; N = 635.

Given\Predict	High	Low
**a**		
High	102	2
Low	4	293
**b**		
High	44	4
Low	4	182
**c**		
High	146	6
Low	8	475

## Discussion

We proposed mbNIR based blood glucose monitoring system applying PMF in SDNN for the first time. The results showed that our sensor model has not only better accuracy but also precision, sensitivity and specificity. Moreover, the introduction of SDNN has made the sensor computationally fast and cheaper than DNN or CNN. It is of particular importance when designing a low-cost blood glucose monitoring system for point-of-care (POC) testing. Blood glucose monitoring systems for POC are regulated, as in the international standard ISO 15197:2013^19^. In brief, two criteria should meet: (1) in comparison to a laboratory method at least 95% of results must be within ± 15 mg/dL at glucose concentrations < 100 mg/dL and within ± 15% at ≥ 100 mg/dL; (2) in a consensus error grid analysis at least 99% of results need to be within zones A and B. Of importance, our sensor model is in compliance with the above criteria’s.

The use of single or multiple-band NIR alone is error prone due to the biometric features such as bones, skin thickness, gender, weights and so on. It can be clearly seen in the Fig. 1a that different individual yielded different NIR/glucose level curves. This could be possibly a reason for previous research groups who tried to impose multiple-band NIR and could not achieve sufficient clinical accuracy with their system due to interference from tissues and bones as shown in Fig. 1b. We encoded PMF into input data and the system was trained applying ten-fold cross validation. As a result, low (approximately 2–6%) drop in the performance of the model was achieved when the performance and training results were compared. The overall (N = 635) accuracy achieved was 97.8% with 96.0% precision, 94.8% sensitivity and 98.7% specificity. On the other hand, an accuracy achieved without PMF was only 60%. It appeared that the two (50 and 30) dense node layers in our model were able to compensate the effect of miniaturizing layers, and thereby enhancing accuracy up to a satisfied level.

Compared to other methods in literature, e.g., Zeng et al. (2013) used mbNIR with single layer Neural Network which is naïve and could not achieve high accuracy^5^. On the other hand, Han et al. (2021) applied complex Deep Neural Networks (DNNs) along with mbNIR and achieve high accuracy^18^. However, we avoid the use of deep layers due to its computational cost involved, and this in turn makes sensor impractical for small devices. Our system instead applied shallow layer with dense nodes (SDNN) in order to form a clear classifier in higher-dimensional hyperplane. Summary of non-invasive approaches was provided in table 2.

**Table 2:** Non-invasive blood glucose approaches in literature.

Non-invasive methods	Part of the body	Advantages	Disadvantages	References
Photoacoustic spectroscopy	Intersitial fluid	Minimally invasive	Need recalibration and require human’s secretion	^20^
Hybrid NIR and GHz wavelength	In vitro (aquas solution)	Improved accuracy compared to barely NIR	Not satisfied clinical accuracy; Further in-invo experiments needed	^6^
Ring resonators	Solution of glucose	Broad glucose range can be measured	Further validation on human required	^21^
photoplethysmogram (PPG) signal + Galvanic skin response	Skin	50 human subject samples reported	Temperature and skin moisture interference Tested only in non-diabetic subjects	^22^
Microwave + Particle Swarm Optimization	Earlobe		High complexity but low accuracy	^23^

## Conclusions

In order to check whether different PMF would result in different characteristic curve between mbNIR vs blood glucose level, we fixed mbNIR but varied PMFs. Finally, we obtained the curves as shown in Fig. 1a. It is shown that characteristic curves were different for each person. As a result, the SDNN model with PMF + mbNIR could be used to predict the personalized characteristic curve of individuals. Hence, our proposed of augmenting PMF into mbNIR for training on SDNN model could predict mbNIR vs glucose curves for each person and enhance the overall performance and accuracy of the system.

For the first time, this work reports two main results: (1) high-precision machine learning regression model for non-invasive blood glucose prediction; (2) excellent DM diagnosis using binary SDNN classification. The proposed sensing model has solved a long-standing research problem of insufficient accuracy using NIR non-invasive glucose sensing. Our sensor model yielded very high performance within 15% error and 95% confidence level for the real-time monitoring of blood glucose in the range of 60–400 mg/dL. Thus, the sensor model complies with ISO 15197:2013, the regulated criteria of blood glucose monitoring system for POC. Since the proposed system is fast and low-cost, it can also be applied for massive DM screening and embedded device for personal blood glucose monitoring. Moreover, the proposed sensing system could globally save lives in Covid-19 outbreaks as DM is one of the main comorbidity factors of Covid-19.

## Methods

### Circuit diagram

**Figure 5:**
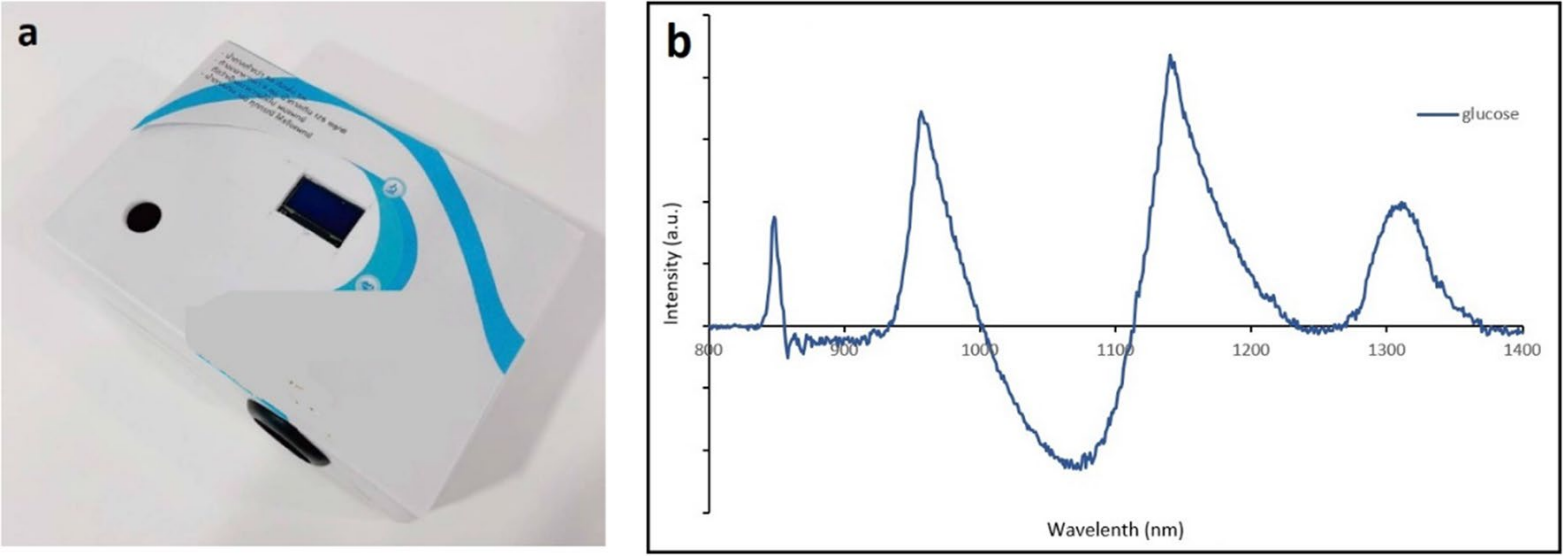
(**a**) The box enclosing the circuits described in Fig. 1. The light is scattered and absorbed in the human finger before entering the photoreceivers of each band. (**b**) Absorbance spectrum of glucose solution.

The non-invasive real-time glucose monitoring system comprises of a box (Fig. 5a), within the box there is a circuit where the details are described by Fig. 2d. The near-infrared LED light sources of wavelengths 850 nm, 950 nm, and 1150 nm were turned on since starting of each measurement, the NIR light were absorbed and scattered through human finger as described by Beer-Lambert law and NIR absorbance characteristic of blood glucose. For each excited frequencies, a photoreceiver of corresponding band was recorded for the scattered intensity. The NIR scattered intensities will then be called multiple-band NIR (mbNIR). The low-passed mbNIR together with PMF were input to SDNN where it computed the blood glucose using the trained neural networks. The process were done in a microcontroller which could be miniaturized inside a box together with the LED transmitter and NIR photoreceivers. The real-time non-invasive glucose predicted value could be displayed on an OLED screen or sending either serially or wirelessly to a computer for making a record.

An absorption and reflectance spectroscopy techniques-based surface mounted device was designed with a multi-band phototransistor array as a near-infrared detector. Stray light blocking filters were introduced to make measurement unaffected in daylight. The sensor components were assembled in a way that the generated mbNIR passes into tissue, gets absorbed and scattered by glucose molecules before hitting a detector (Fig. 2c,d). The output was obtained serially in frames corresponding to each sample.

### The shallow dense neural network architecture

The proposed NN structure were shallow in the sense that it was computationally fast compared to deep neural networks. The input datasets namely mbNIR and PMF were placed at the beginning of the SDNN, followed by the first and second layers of dense 50 and 30 nodes, respectively. MbNIR data obtained was shown in Fig. 2a. Absorbance spectrum of glucose solution was shown in Fig. 5b. Figure 2a illustrates a design of the SDNN model. The number of the first hidden layers were 50 nodes in order to encode information from the input data of 9 fields into higher-dimensional space. The number of hidden layer nodes was obtained from adjusting in order to optimize the training performance using the learning curve vs number of nodes. The minimal number of nodes in the first hidden layer which could achieve the same performance would be 50 nodes at least. For the second hidden layer of 30 nodes, it is decoding information from higher-dimensional space before the information was sent to the decision node. The number of second hidden layer was obtained experimentally using hyperparameter tuning. Sigmoid functions were used as the activation function in the two hidden dense layer nodes. In the last node, ReLU activation function was used to numerically predict the real-time blood glucose. Randomized weight matrix initialization has been used.

### Study subjects

The training and evaluation of the sensor model was performed in general population consisting of healthy, diabetes and undiagnosed diabetes. The only criterion for subject’s inclusion in the study was adult aged ≥ 18 years. A dataset of 401 subjects were applied as a training set. Additionally, a cohort of 234 individuals not included in the model training set were used to evaluate the performance of the model. They were aged 27–69 years and the part of chronic kidney disease screening sub-project under the Chronic Kidney Disease Prevention in the Northeast of Thailand^24^. The study protocol conforms to the ethical guidelines of the 1975 Declaration of Helsinki that was approved by the Ethics Committee for Human Research, Faculty of Medicine, Khon Kaen University, Thailand (project number HE 601,035) and all participants provided written informed consents.

### The reference method for blood glucose

We measure plasma glucose level in order to validate the performance of our developed non-invasive glucose sensor. The technique was based on Hexokinase method using Roche Cobas 8000. The method works in principle that glucose molecule in the sample reacts with ATP in the presence of Hexokinase to form glucose-6-phosphate. Glucose-6-phosphate (G-6-P) is then converted into gluconate-6-phosphate via G-6-P dehydrogenase in the presence of NADP^+^. The reduction of NADP^+^ into NADPH results in an increment of absorbance peak at 340 nm and 700 nm, which is directly proportional to the blood glucose level.
